# The epicardium obscures interpretations on endothelial-to-mesenchymal transition in the mouse atrioventricular canal explant assay

**DOI:** 10.1038/s41598-018-22971-w

**Published:** 2018-03-16

**Authors:** Nathan Criem, An Zwijsen

**Affiliations:** 1 VIB-KU Leuven Center for Brain & Disease Research, KU Leuven, Belgium; 2 Department of Human Genetics, KU Leuven, Belgium; 3 Present Address: Center for Molecular and Vascular Biology, Department Cardiovascular Research, KU Leuven, Belgium

## Abstract

Atrioventricular septal defects often result from impaired endocardial cushion development. Endothelial-to-mesenchymal transition (EndoMT) is a critical event in endocardial cushion development that initiates in the atrioventricular canal (AVC). In *ex vivo* EndoMT studies, mouse AVCs are flat-mounted on a collagen gel. In the explant outgrowths, the ratio of elongated spindle-like mesenchymal cells over cobblestone-shaped cells, generally considered as endothelial cells, reflects EndoMT. Using this method, several key signalling pathways have been attributed important functions during EndoMT. Using genetic lineage tracing and cell-type-specific markers, we show that monolayers of cobblestone-shaped cells are predominantly of epicardial rather than endothelial origin. Furthermore, this epicardium is competent to undergo mesenchymal transition. Contamination by epicardium is common and inherent as this tissue progressively attaches to AVC myocardium. Inhibition of TGFβ signalling, previously shown to blunt EndoMT, caused an enrichment in epicardial monolayers. The presence of epicardium thus confounds interpretations of EndoMT signalling pathways in this assay. We advocate to systematically use lineage tracers and cell-type-specific markers on stage-matched AVC explants. Furthermore, a careful reconsideration of earlier studies on EndoMT using this explant assay may identify unanticipated epicardial effects and/or the presence of epicardial-to-mesenchymal transition (EpiMT), which would alter the interpretation of results on endothelial-to-mesenchymal transition.

## Introduction

To safeguard a unidirectional blood circulation, the heart develops the mitral and tricuspid valves in the atrioventricular canal (AVC) and the semilunar valves in the outflow tract (OFT)^1^. In mice, heart valve development initiates with an increased deposition of extracellular matrix, the cardiac jelly, between the myocardial and endothelial components of the AVC and OFT around embryonic day (E)9.0. This leads to swellings known as cardiac cushions. In the AVC, myocardium-secreted ligands such as BMP2 (bone morphogenetic protein 2) and TGFβ2 (transforming growth factor *beta* 2) then trigger the adjacent endothelium to undergo an endothelial-to-mesenchymal transition (EndoMT) between E9.0 and E9.5^2–5^. In the OFT cushions, the majority of the mesenchymal cells are, however, derived from neural crest cells^6^. The newly formed mesenchymal cells invade the cardiac jelly and proliferate.Subsequently,  the cushions progressively remodel into valve leaflets from E12.5 onwards^7,8^.

In 1979, Bernanke and Markwald established a chicken AVC explant assay to study EndoMT *ex vivo*^9,10^. In this assay, AVCs are cultured on collagen gels for several days. The endothelial cells grow out from the explant and invade the gel as mesenchymal cells. This assay was later adapted for mouse embryos^4,11–13^. In 2002, Camenisch *et al*.^4^, reported maximal EndoMT in cultures isolated from E9.5 staged mouse embryos between 20–26 somites (s) and the increasing presence of cobblestone-shaped endothelial cell monolayers from 26 s onwards. Since then, the AVC explant assay, in combination with genetic mouse models, has proven to be a powerful tool to study the molecular mechanisms regulating EndoMT^4,8,14–18^.

While establishing this assay, we observed the typical somite stage-dependent pattern in cellular outgrowth as previously reported^4,5^, including the cobblestone-shaped monolayers that were present from 23 s–26 s onwards. However, these monolayers were remarkably devoid of the endothelial marker CD31. Combining endothelial lineage tracing with immunofluorescent marker analyses, we show that an increasing number of epicardial cells contribute to the explant outgrowths. These epicardial cells migrate as monolayers, thereby resembling endothelial cell monolayers, and are often positive for *alpha* Smooth Muscle Actin (αSMA), suggestive of epicardial-to-mesenchymal transition (EpiMT) competence.

Inhibition of TGFβ-signalling resulted in an enrichment in cobblestone-shaped monolayers, identified earlier as endothelium^4^, yet the majority of these monolayers were positive for Wilm’s Tumor Protein1 (WT1), compatible with an epicardial origin. These data highlight that a stringent lineage control and somite-staging is critical to preclude confounding data interpretation in this established assay for EndoMT.

## Results

**Figure 1:**
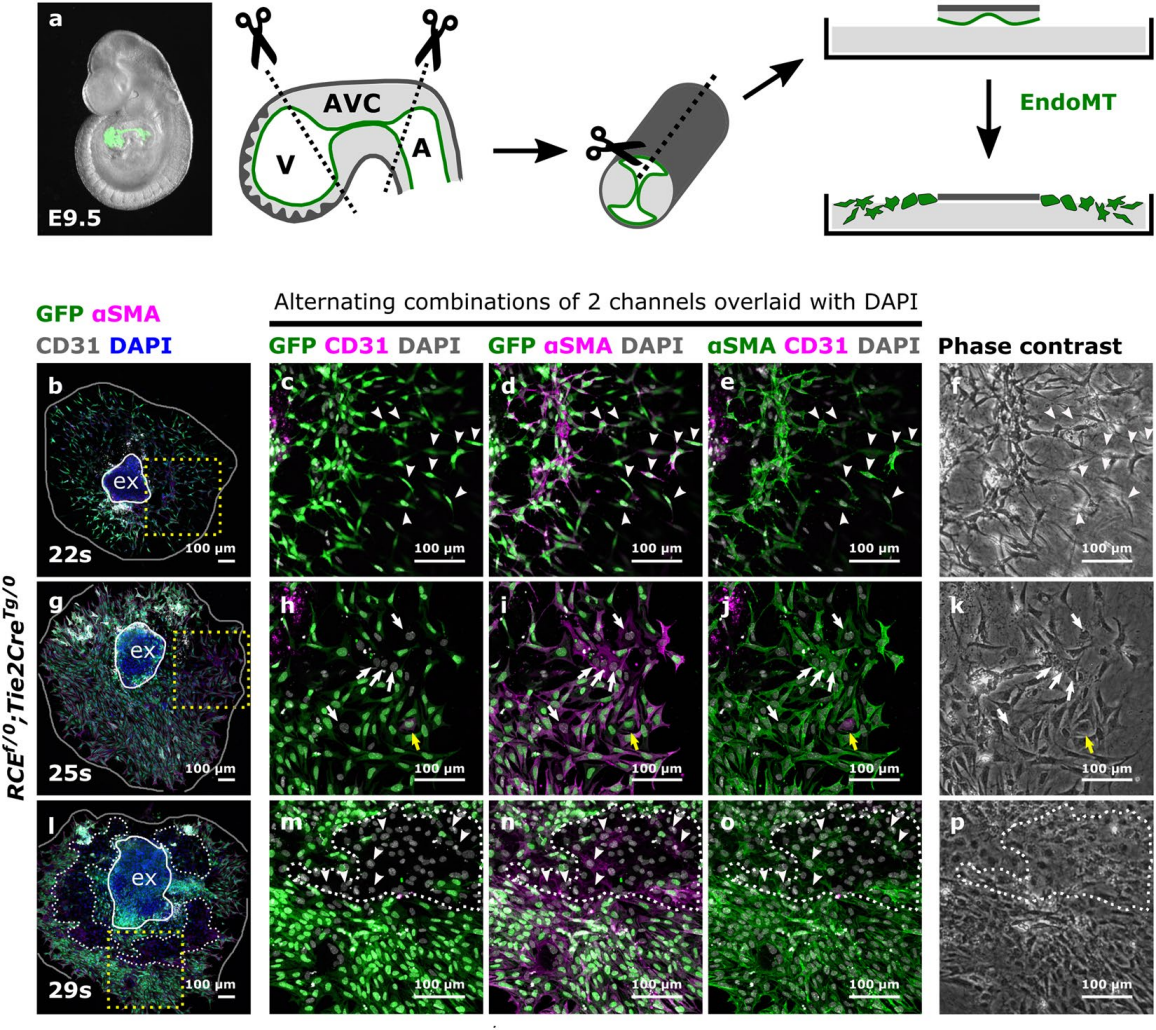
The cellular outgrowth pattern in mouse AVC explants differs depending on the somite-stage of the embryo. (**a**) Schematic representation of the mouse AVC explant assay. The endocardium from an E9.5 embryo is highlighted in green. The AVC is marked by the “kissing zone” and the cushions. (**b**–**p**) AVCs collected from *RCE*^*f/0*^*;Tie2Cre*^*Tg/0*^ embryos staged between 22 s–34 s were cultured for 48 h, immunostained for GFP, αSMA and CD31 and imaged by confocal microscopy (N = 19 explants). Boxed areas in (**b**), (**g**) and (**l**) are magnified in respectively (**c**–**e**), (**h**–**j**) and (**m**–**o**) and presented as alternating combinations of two channels in magenta and green, overlaid with DAPI in gray. Individual confocal Z-planes are compared with the corresponding phase contrast images (**f**,**k** and **p**). The explant (ex) is delineated by a white loop. The area of cellular outgrowth in panels (**b**), (**g**) and (**l**) is delineated by a gray loop. White dashes in panels (**l**–**p**) indicate GFP-negative monolayers. White arrowheads in panels (c–f) indicate αSMA^+^GFP^+^CD31^−^ spindle-like shaped mesenchymal cells. White arrowheads in panels (m–o) indicate αSMA^+^GFP^-^CD31^−^ cobblestone-shaped cells. The yellow arrows in panels (h–j) indicate an αSMA^+^GFP^+^CD31^+^ endothelial cell. The white arrows in panels (h–j) indicate αSMA^+^GFP^-^CD31^-^ cobblestone-shaped cells. A: atrium, AVC: atrioventricular canal, EndoMT: endothelial-to-mesenchymal transition, V: ventricle.

To determine the origin of the cellular outgrowths in AVC explants, Cre-reporter (*RCE*^*f/f*^) females were crossed with *Tie2Cre*^*Tg/0*^ males. *Tie2Cre*^*Tg/0*^*;RCE*^*f/0*^ embryos were collected between E9.0-E10.5 and somite staged for AVC cultures (Fig. 1a). In such embryos all endothelial cells and their derivatives are GFP-positive. Consistent with previous reports^4,15^, a somite-stage dependent outgrowth pattern was observed. Between 20 s–23 s, almost all cells grew out from the explants as spindle-like shaped mesenchymal cells indicating that EndoMT was effective (Fig. S1a). From 23 s–26 s onwards, increasing amounts of cobblestone-shaped cells became apparent (Fig. S1b) and from 26 s onwards, all explant outgrowths contained monolayers of cobblestone-shaped cells (Fig. S1c–d). These cobblestone-shaped cells and monolayers are qualified in literature as endothelial cells resulting from reduced EndoMT from this stage onwards^4,15^.

**Figure 2:**
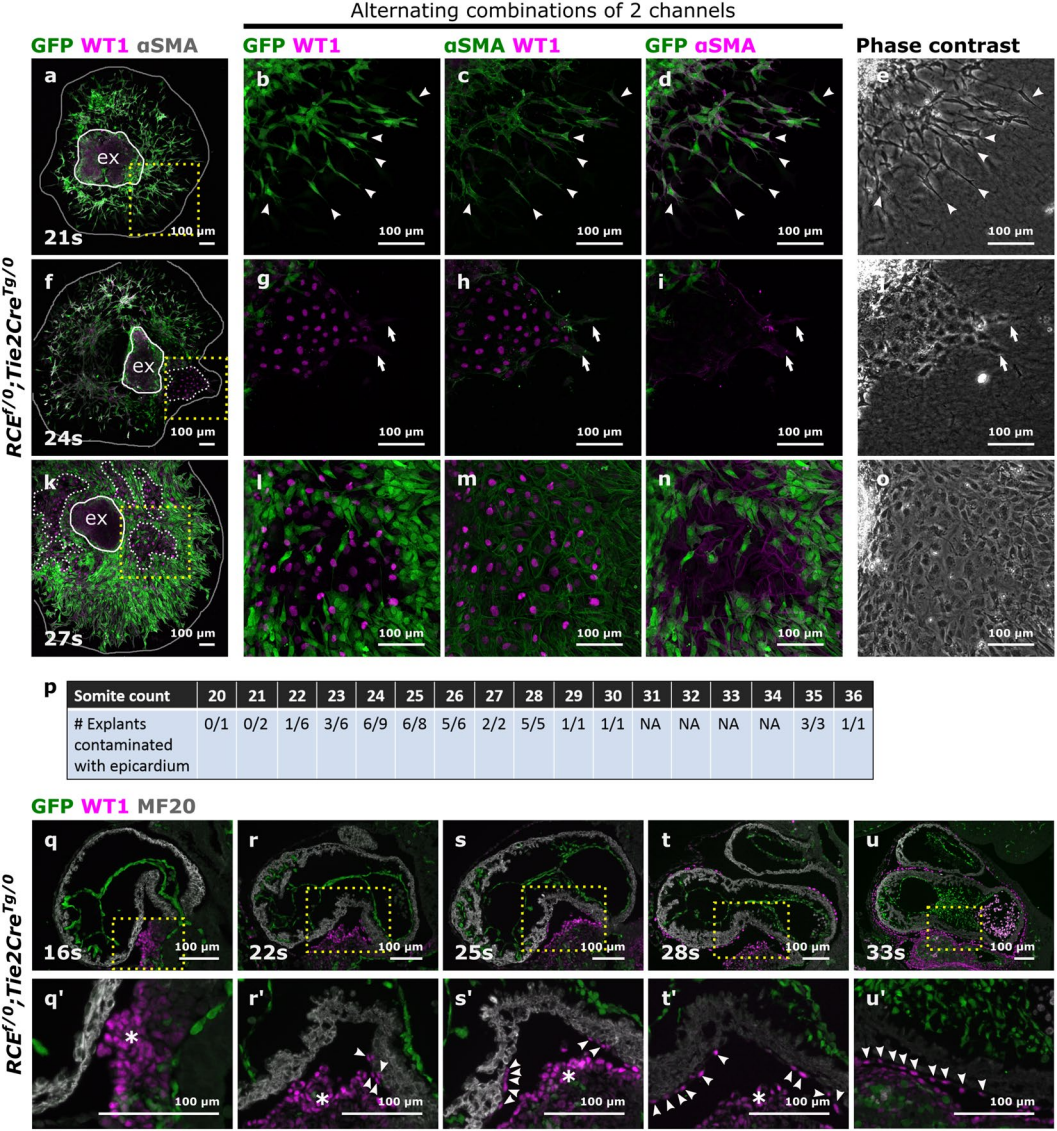
Epicardial cells contaminate the AVC explant with increasing somite stage which corresponds with the progressive covering of the myocardial wall by epicardial cells. (**a**–**o**) AVCs collected from *RCE*^*f/0*^;*Tie2Cre*^*Tg/0*^ embryos staged between 20 s–36 s (N = 53 explants) were cultured for 48 h and stained for GFP, WT1 and αSMA. Boxed areas in (**a**), (**f)** and (**k**) are magnified in respectively (**b**–**d**), (**g**–**i**) and (**l**–**n**) and presented as alternating combination of 2 channels in magenta and green. Individual confocal Z-planes are compared with the corresponding phase contrast images (**e**, **j** and **o**). The explant (ex) is delineated by a white loop. The area of cellular outgrowth in panels (a), (f) and (k) is delineated by a gray loop. Arrowheads in panels (b–e) indicate αSMA^+^GFP^+^WT1^−^ spindle-like shaped mesenchymal cells. Arrows in panels (g–j) indicate epicardial cells undergoing EpiMT. (**p**) Table representing the numbers of explants isolated per somite stage and the number of explants contaminated with epicardium. NA: not applicable, not dissected in this experiment. (**q**–**u**) Parasagittal sections from mouse embryos collected between 16 s–35 s (N = 24 embryos) were stained for GFP, WT1 and MF20. Boxed areas are magnified in (q’-u’). Arrowheads in panels (q’-u’) indicate epicardial cells migrating along the myocardial wall. Asterisks indicate the pro-epicardial organ.

To verify the identity of the migrating cells, the explants were immunostained for GFP, the endothelial marker CD31 and the mesenchymal marker αSMA (Figs 1b–p, S1e–l). Between 20 s–23 s, most spindle-like shaped cells were, as expected, GFP^+^αSMA^+^CD31^−^ mesenchymal cells (Fig. 1b–e). Sporadically, GFP^+^αSMA^+^CD31^+^ cells were detected, compatible with endothelial cells undergoing EndoMT (Fig. 1g–j). A key aspect of the AVC explant assay is the invasion of mesenchymal cells into the collagen gel. Invasiveness was maximal between 20 s–23 s and decreased between 23 s–26 s with the emergence of monolayers (Fig. 2S). Little to no invasion was observed from 27 s–30 s onwards (data not shown). Remarkably, the monolayers of cobblestone-shaped cells observed from 26 s onwards were almost always devoid of GFP and CD31 staining, with a few occasional cells being positive for αSMA (Fig. 1l–o, arrowheads). The presence of αSMA and absence of CD31 suggested that these cells initiated mesenchymal transition but had not yet detached from each other as has previously been suggested for explants cultured in high glucose conditions^19^. The absence of GFP could indicate a failure of Cre-mediated recombination in some cells. This is, however, unlikely as the *Tie2Cre* line contains a constitutively active Cre-recombinase and the number of GFP-negative cells consistently increased from 26 s onwards. Therefore, we concluded that the cobblestone-shaped monolayers were of non-endothelial cell origin.

In mice, the pro-epicardial organ attaches to the heart around E9.5. Epicardial cells progressively envelop the heart and start invading the myocardium through EpiMT and later contribute to the coronary vasculature and cardiac fibroblasts^20–23^. To determine whether the observed monolayers in the AVC explants were epicardium-derived, *Tie2Cre*^*Tg/0*^*;RCE*^*f/0*^ explants were immunofluorescently stained for GFP, αSMA and the epicardial marker WT1^20^ (Fig. 2a–o). From 20 s–22 s embryos, nearly all explant outgrowths were devoid of WT1-positive cells (Fig. 2a–d,p). Conversely, the number of explants containing WT1-positive monolayers considerably increased from 23 s–26 s onwards (Fig. 2f–p). This supported our hypothesis that the observed monolayers were of epicardial origin. Furthermore, we sporadically observed individual αSMA^+^GFP^−^WT1^−^ cells (Fig. f-j) that grew from the monolayers as spindle-like shaped cells, compatible with the competence of epicardial cells to undergo EpiMT^21,22^. Confocal optical sections within the collagen gel only showed GFP-positive endothelium-derived invasive mesenchymal cells (Fig. S2e–h). Overall, these observations illustrate that the assessment of cell shape alone is insufficient to categorize the different cell types.

To test whether the epicardium is already covering the AVC of the heart at the stages used for the AVC explants, tissue sections from *Tie2Cre*^*Tg/0*^*;RCE*^*f/0*^ mouse embryos were immunofluorescently stained for WT1, GFP and the myocardial marker MF20. Physical contact between the pro-epicardial organ and the heart was present as early as 16 s (Fig. 2q). Increasing attachment of WT1^+^ epicardial cells on the AVC myocardium was detected from 20 s–22 s onwards (Fig. 2r–u). This demonstrates that epicardial cell contamination in AVC explants is inherent to the technique and not due to a dissection artefact. The near absence of epicardial cells in explants collected before 23 s likely reflects that a critical threshold of epicardial cells is required to compete with the endothelial outgrowth.

**Figure 3:**
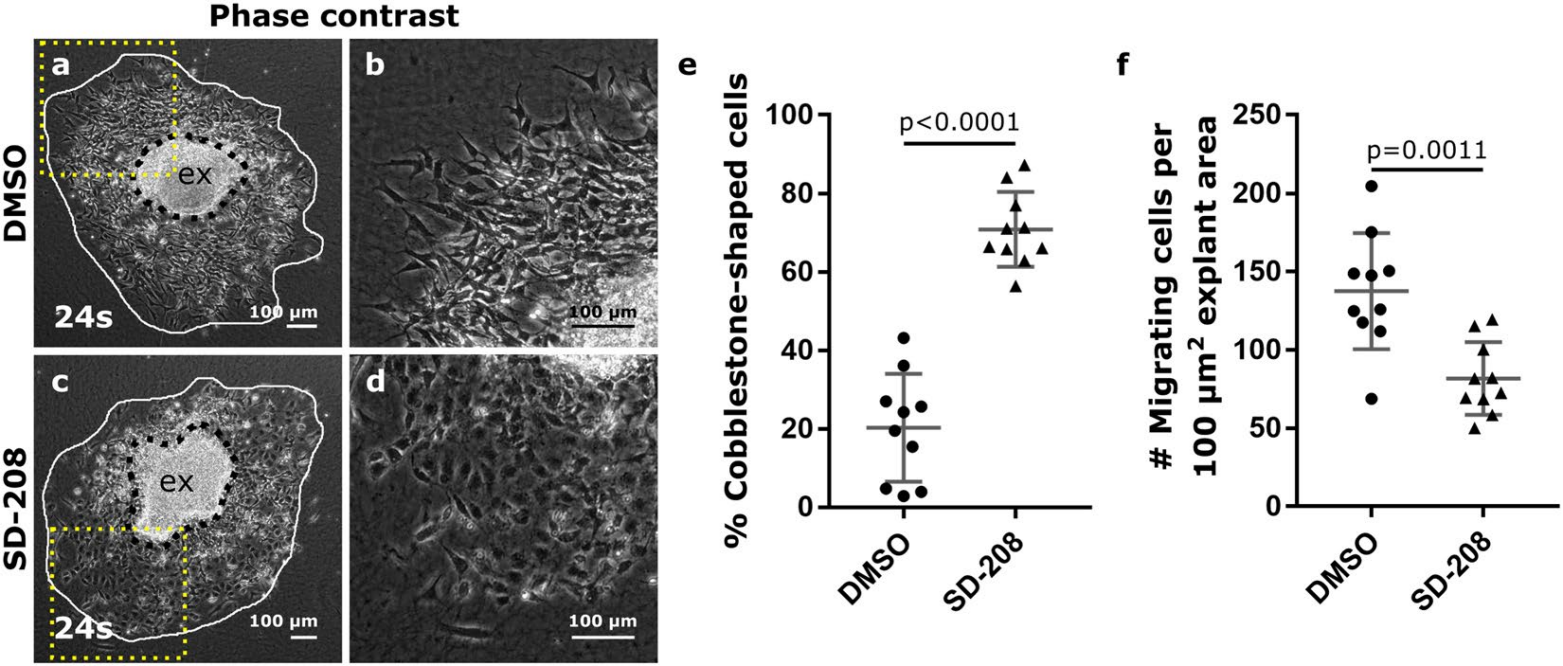
Inhibition of ALK5 leads to an enrichment in cobblestone-shaped cells. Phase contrast figures of control DMSO (**a**,**b**) and SD-208 (**c**,**d**) treated explants. Boxed areas in panels (a) and (c) are magnified in panels (b) and (d) respectively. (**e**) Quantification of the relative amount of cobblestone-shaped cells in the outgrowths. (**f**) The total number of migrating cells normalized to the explant area (presented per 100 µm^2^ explant area). The individual datapoints, the mean and standard deviation are presented in panels (e) and (f). The explants (ex) is delineated by black dashes. The area of cellular outgrowth is delineated by a white loop.

**Figure 4:**
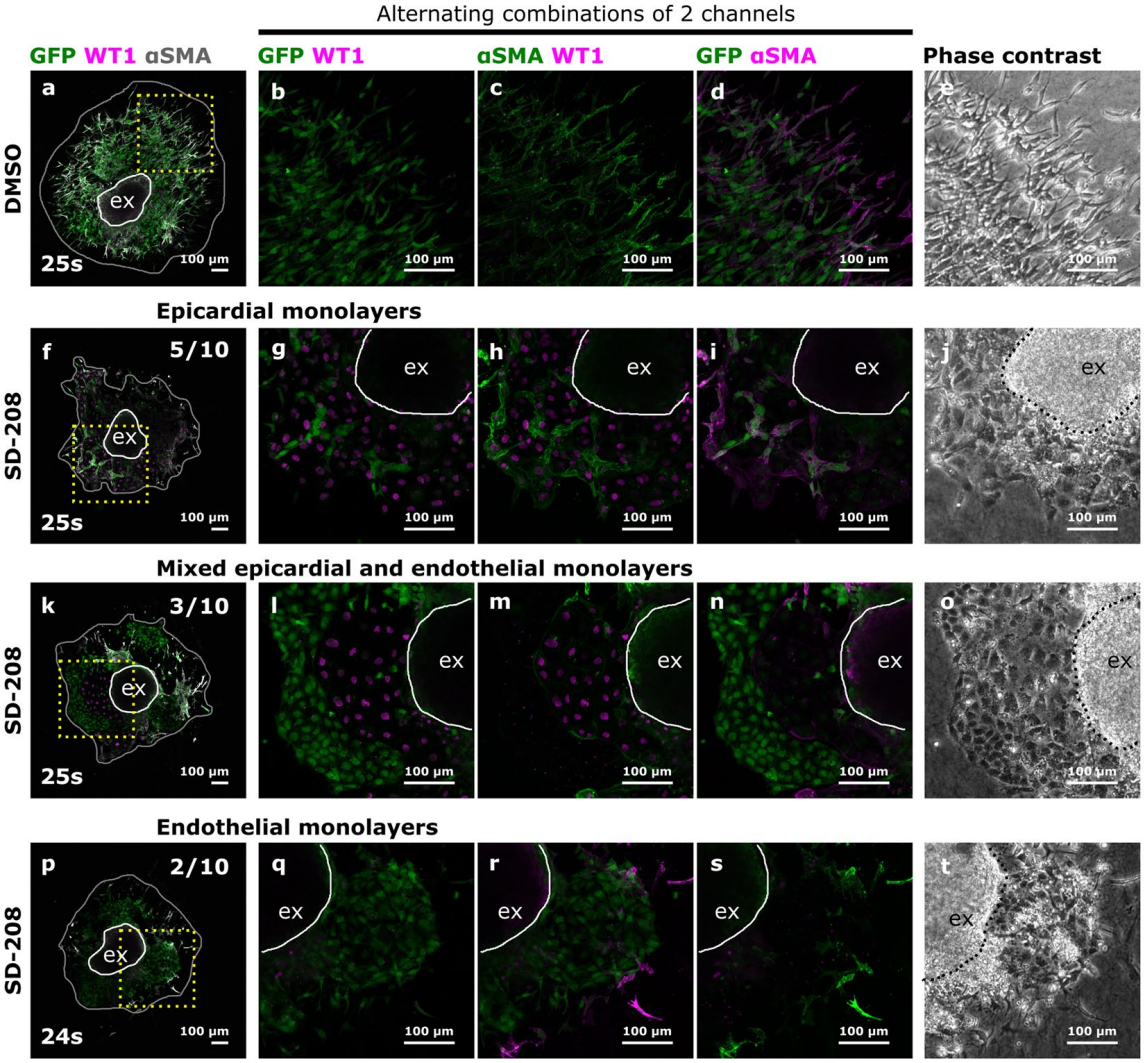
Inhibition of ALK5 leads to a relative increase in epicardial and endothelial cells. (**a**–**t**) 10 pairs of somite matched AVC explants treated with either DMSO or SD-208 in DMSO were stained for GFP, WT1 and αSMA. Boxed areas in panels (a), (f), (k) and (p) are magnified in respectively (**b**–**d**), (**g**–**i**), (**l**–**n**) and (**q**–**s**) and presented as alternating combinations of 2 channels in magenta and green. Individual confocal Z-planes are compared with the corresponding phase contrast images (**e**,**j**,**o** and **t**). The explant (ex) is delineated by a white loop. The area of cellular outgrowth in panels (a), (f), (k) and (p) is delineated by a gray loop. Upon SD-208 treatment, the observed monolayers are either epicardium only (**f**–**j**), endothelium only (**p**–**t**) or mixed epicardium and endothelium (**k**–**o**).

The AVC explant assay has been used in many studies to investigate whether specific signalling pathways are involved in EndoMT^4,11,14,15,24^. It was previously shown that inhibition of TGFβ2 signalling in AVC explants results in an enrichment of cobblestone-shaped cells^4^. Based on our findings, we questioned whether these cobblestone-shaped cells may have been of epicardial origin, thereby confounding the interpretation on EndoMT. To address this question, we inhibited ALK5, a type I TGFβ receptor, using SD-208^25^. Explants from somite-matched embryos (24 s–27 s) were treated with either SD-208 or DMSO as a vehicle control. Decreased pSMAD3 immunostaining confirmed the effective reduction in TGFβ signalling in SD-208 treated samples (Fig. S3). In line with previous studies^4,26^, ALK5 inhibition led to a relative enrichment in cobblestone-shaped cells in monolayers and a reduction in the number of migrating cells (Fig. 3) However, lineage tracing and marker analysis revealed the presence of two distinct types of monolayers; αSMA^+^GFP^-^WT1^+^ epicardial monolayers (Fig. 4f–j) and αSMA^−^GFP^+^WT1^−^ endothelial monolayers (Fig. 4p–t). Both types of monolayers could be present either alone or in combination upon ALK5 inhibition (Fig. 4k–o). Interestingly, epicardial cells were notably larger in size than endothelial cells and displayed a more prominent nucleus. This observation demonstrates that in our study, and perhaps also in others, the presence of cobblestone-shaped monolayers may be due to epicardium contamination rather than failure in EndoMT alone.

## Discussion

**Figure 5:**
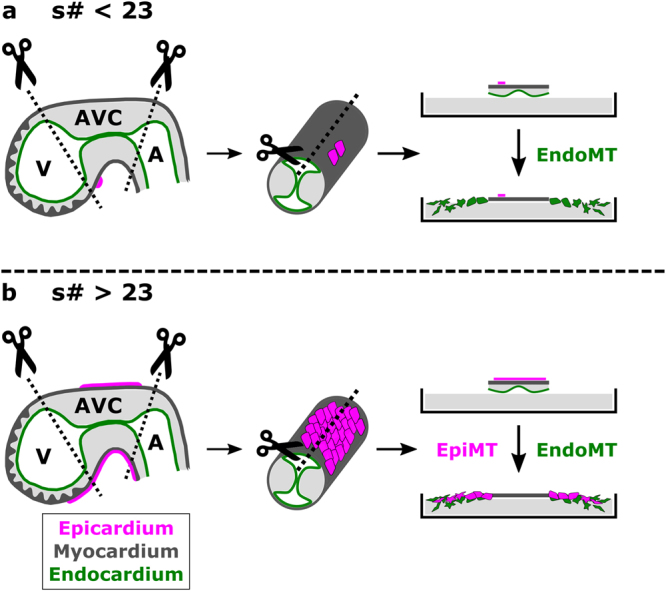
Schematic representation of the AVC explant model with and without epicardial cell contamination. (**a**) Explants collected from embryo between 20 s–23 s contain none to nearly no epicardial cells. Only endothelium-derived mesenchymal cells will grow out of the explant. (**b**) From 23 s onwards, epicardial contamination increases and becomes apparent as monolayers of epicardium-derived cells capable of undergoing epicardial-to-mesenchymal transitions.

With the present study, we aim to raise awareness on epicardial cell contamination in the mouse AVC explant assay and how this may confound interpretations of results regarding EndoMT. Using lineage tracing in combination with marker analyses, we show that cobblestone-shaped morphology in AVC outgrowths is insufficient to qualify cells as endothelial, and that mesenchymal cells in explants can be obtained via EndoMT and EpiMT processes (Fig. 5).

A previous study briefly acknowledged that epicardial cells may contaminate explants collected from 30 s onwards^5^. To minimize epicardial cell contamination, the authors scraped the outside wall of 30 s–35 s AVC explants with a tungsten needle, but not those of 20 s–29 s explants. Despite this treatment, the explants still contained monolayers morphologically reminiscent to the WT1-positive monolayers in our study which questions the effectiveness of the epicardial removal and the conclusion that were drawn by the author.

The fact that epicardial cells contaminate and contribute to AVC explant outgrowths has multiple implications. Several key publications in the field of heart valve development have used the AVC explant assay as supporting evidence to attribute regulatory functions to TGFβ, vascular endothelial growth factor (VEGF) and other signalling pathways on EndoMT based on evaluation of cell shape in AVC explant outgrowths^4,5,11,24^. Our present data now challenges the interpretations of these studies. For example, inhibition of TGFβ2 was shown to inhibit EndoMT as assessed by the enrichment in cobblestone-shaped monolayers^4^. In our hands, these monolayers were predominantly of epicardial origin. Whether epicardial, endothelial or mixed epicardial/endothelial monolayers grow out may depend on the relative coverage of the myocardial wall by epicardium. Furthermore, the extent of damage that is inflicted on the endothelial and epicardial cells during the procedure may balance the outcome of which type of monolayer emerges. Interestingly, cultures of pro-epicardial organs have been shown to undergo EpiMT when stimulated with VEGF, TGFβ1 or TGFβ2^27^. This is of particular relevance when working with AVC explants from E10.5 embryos as they are prone to epicardial cell contamination.

Besides confounding interpretations on cell identity, the epicardium is also a source of factors that may on their turn interfere with the process of EndoMT (e.g. retinoic acid^28^). This adds yet another layer of variability and complexity to this assay. Our data demonstrate that caution must be taken when performing experiments with explants isolated from embryos above 23 s–26 s as epicardial rather than endocardial or endocardial-derived cells may be studied.

We recommend to use explants from embryos staged between 20 s–23 s to minimize the risk of epicardial cell contamination and to systematically compare carefully somite-matched explants when gain- and loss-of-function experiments are performed. Such a narrow somite-staged pairing does make experiments challenging due to the high variability in somite stages among littermates. Because the stage-dependent dynamics of EndoMT and epicardial outgrowth in AVC explants may differ between different genetic backgrounds, standard probing of explants for epicardial cell contamination using lineage tracing and marker analyses is recommended. The selection of appropriate markers is critical albeit not trivial. Indeed, additional (single) stainings for phalloidin^11,14,29^ or IsolectinB4^16^ have sometimes complemented AVC outgrowth analyses. Phalloidin yields information on cell shape but cannot differentiate epicardial- from endothelial-derived mesenchyme. The use of isolectinB4 as an endothelial marker is inconclusive in the AVC assay as we recently found that it also marks the epicardium in tissue sections^30^. We provide here an immunostaining toolkit to distinguish WT1+ epicardium from CD31+ endothelium in AVC explants collected from 20 s to 35 s. Finally, we advocate a careful reinterpretation of available literature on the AVC explant models and EndoMT when a thorough marker analysis is lacking.

## Materials and Methods

### Experimental animals

*RCE*^*f/f*^ females^31^ and *Tie2Cre*^*Tg/0*^ males^32^, both on a CD1 background, were mated. The presence of a vaginal plug was considered as embryonic day (E)0.5 at noon. Pregnant females were sacrificed 9 or 10 days later to collect embryos. All mouse experiments were approved by the ethical committee of the Animal Research Centre of the KU Leuven, Belgium (project file number P209/2013). Experiments were conducted in accordance with the legislation.

### Collagen gel preparation

Rat tail type 1 collagen gels (Merck 08–115) were prepared at 1 mg/ml in 10 × M199 (Life Technologies 11825–015) and the pH was adjusted to 7.5 with 2.2% (w/v) NaHCO_3_. 10 × M199 and NaHCO_3_ were both measured at 1/10 of the final volume. The final collagen solution was dispensed at 400 µl per well in 12-well plates (Greiner) and allowed to polymerize for 1 h at room temperature before washing twice for 5 min with Opti-MEM (Invitrogen 31985047). Next, the gels were incubated overnight at 37 °C with an equal volume (400 µl) of Opti-MEM supplemented with 1% fetal calf serum (Lonza), 50 µg/ml streptomycin (Life Technologies 15140122), 50 U/ml penicillin (Life Technologies 15140122), 2 mM L-glutamine (Life Technologies 25030-024) and 0.1% ITS (insulin, transferrin, selenium, Life Technologies 51500-056). Culture medium was removed just prior to the dissection of the explants.

### Atrioventricular canal explants

Embryos of E9.0 to E10.5 were dissected in Tyrode’s salt solution (Sigma Aldrich T2397) and staged by counting the number of somite pairs. GFP positivity was verified using a Zeiss Discovery V12 dissection microscope (InfraMouse: Hercules ZW09-03). Next, the pericardium was removed, the heart excised and the AVC was identified by the cushion “kissing zone” (Fig. 1A). AVCs were microdissected by first pinching off the ventricle and subsequently the primitive atrium. It is imperative to expose the endothelium to the collagen to maximize EndoMT and minimize epicardial cell contamination. Thereto, the AVCs were longitudinally opened and residual ventricle or atrium was trimmed off. Each AVC was transferred to an individual gel by pipetting and residual liquid was removed. Using forceps, the AVC explant was positioned centrally on the gel with the endothelium facing downwards. This procedure was finalized within the hour following euthanasia of the dam. After attachment for 10 h at 37 °C and 5% CO_2_, 150 µl of culture medium was added per gel (M199, Life Technologies 11150-059) supplemented with 1% fetal calf serum, 50 µg/ml streptomycin, 50 U/ml penicillin, 2 mM L-glutamine and 0.1% ITS. Culture medium can be added after 2 hours of attachment although a longer period is recommended when preparing explants collected from mutant embryos with impaired EndoMT as they may detach more easily. The explants were cultured for 48 h at 37 °C and 5% CO_2_ and fixed for 10 min with freshly prepared MEMFA solution (3.7% formaldehyde, 100 mM MOPS (4-Morpholinepropanesulfonic acid), 2 mM EGTA, 1 mM MgSO_4_, pH 7.4). After fixation, the explants were washed with TBS (150 mM NaCl, 150 mM Tris, pH7.5) and stored in TBS with 0.1% NaN_3_ at 4 °C until further processing.

### ALK5 inhibition

AVCs were explanted and allowed to attach for 2 hours after which culture medium with a final concentration of 3 µM SD-208 (Sigma-Aldrich S7071) and 0.1% dimethyl sulfoxide (DSMO, Sigma-Aldrich D5879) was added. In controls, 0.1% DMSO in culture medium was used. The AVCs were cultured for 48 h.

### Data availability

The dataset generated for Fig. 3 is available from the corresponding author on reasonable request.

### Immunofluorescent stainings on AVC explant

The explants were washed with TBS, quenched with 50 mM NH_4_Cl for 30 min at RT, washed again with TBS and blocked for 1 h with 3% BSA (bovine serum albumin, Sigma A9647) in TBS with 10% normal donkey serum (Jackson ImmunoResearch 017-000-121) and 0.3 M glycine. The explants were then sequentially stained for WT1 (Abcam ab89901, diluted 1/500), αSMA (Sigma-aldrich A2547, diluted 1/250) and GFP (Abcam ab13970, diluted 1/500) in the mentioned order or stained for CD31 (BD 553371, diluted 1/250), αSMA and GFP or pSMAD3 (Abcam ab52903, diluted 1/200). WT1 presence is restricted to epicardium until E12.5 in the mouse heart^33^. All primary antibodies were diluted in 3% BSA/TBS-T (TBS, 0.1% Tween20) and incubated overnight at 4 °C. Explants were washed with TBS-T from the primary antibody incubation onwards. Secondary antibodies (Jackson ImmunoResearch, diluted 1/500) were: donkey anti-rabbit-Cy3 (Jackson ImmunoResearch 711-165-152), donkey anti-mouse-Cy3 (Jackson ImmunoResearch 715-165-150), donkey anti-mouse-alexa647 (715-606-151) and donkey anti-chicken-alexa488 (703-546-155). For CD31 detection, the blocking step was preceded by a 10 min incubation with 3% H_2_0_2_ in methanol followed by TBS washes. The antibody staining was amplified using the Perkin Elmer TSA NEL700A001KT kit and detected with streptavidin-alexa647 (Jackson ImmunoResearch 016-600-084). The tyramide was diluted 1/100 in amplification diluent. Streptavidin-HRP (Perkin Elmer TSA NEL700A001KT), secondary antibodies and streptavidin-Alexa647 were diluted 1/500 in 3% BSA/TBS-T and incubated for 30 min at RT. DAPI (Life Technologies D3571, diluted 1/1000 in TBS) was used as nuclear counterstain. Confocal images were acquired using a Nikon A1R Ti Eclipse and corresponding NIS-elements software. An individual Z-slice is shown for each explant. Phase contrast images were acquired using a Leica DMI 6000 B microscope and corresponding LAS software.

### Immunofluorescent stainings on paraffin section

*Tie2Cre*^*Tg/0*^*;RCE*^*f/*+^ E9.0-E10.5 embryos were dissected in ice-cold PBS, fixed overnight in 4% paraformaldehyde in PBS, embedded in paraffin and para-sagittally sectioned at 6 µm thickness.

The slides were deparaffinized in xylene and rehydrated in a decreasing ethanol series. For antigen retrieval, the slides were immersed in citrate-buffer (10 mM Tri-Sodium Citrate dihydrate, 0.01% Tween20, pH6) for 40 min at 96 °C followed by a 20 min cool-down at 4 °C. The sections were blocked with 3% BSA in TBS and incubated overnight with antibodies against WT1 (Abcam ab89901, diluted 1/200) and GFP (Abcam ab13970, diluted 1/200) in 3% BSA in TBS-T. The next day, the slides were washed with TBS-T and incubated with anti-rabbit-Cy3 and donkey anti-chicken-alexa488 for 1 h at RT. The slides were washed and incubated overnight at 4 °C with an antibody against MF20 (R&D MAB4470, diluted 1/50). Biotinylated donkey anti-mouse (Jackson ImmunoResearch 715-065-150) was used as secondary antibody followed by a streptavidin-alexa647 (Jackson ImmunoResearch 016-600-084, diluted 1/100). Secondary antibodies were diluted 1/300 in 3% BSA in TBS-T. DAPI was used to stain the nuclei. Images were acquired using a Leica DM5500 fluorescent microscope and corresponding LAS AF imaging software (InfraMouse: Hercules ZW09-03).

### Statistics

Sample sizes were not pre-determined. Normal distribution of the dataset in Fig. 3 was verified and confirmed using Shapiro-Wilk and D’Agostino & Pearson normality tests. A Welch’s t test was used to determine statistical significance. A p-value below 0.05 was considered statistically significant. GraphPad Prism 7.02 was used for statistical calculations.

### Image processing

FIJI (Fiji is just ImageJ), GIMP (GNU image manipulation program) and Inkscape were used for image processing.

## Electronic supplementary material

Supplementary Figures
