# Author Correction: The epicardium obscures interpretations on endothelial-to-mesenchymal transition in the mouse atrioventricular canal explant assay

**DOI:** 10.1038/s41598-020-60109-z

**Published:** 2020-03-31

**Authors:** Nathan Criem, An Zwijsen

**Affiliations:** 1 VIB-KU Leuven Center for Brain & Disease Research, KU Leuven, Belgium; 2 Department of Human Genetics, KU Leuven, Belgium; 3 Present Address: Center for Molecular and Vascular Biology, Department Cardiovascular Research, KU Leuven, Belgium

Correction to: *Scientific Reports* 10.1038/s41598-018-22971-w, published online 16 March 2018

In Figure 4, the panels r and s were presented as s and r respectively. The correct Figure 4 appears below as Figure 1.

**Figure 1:**
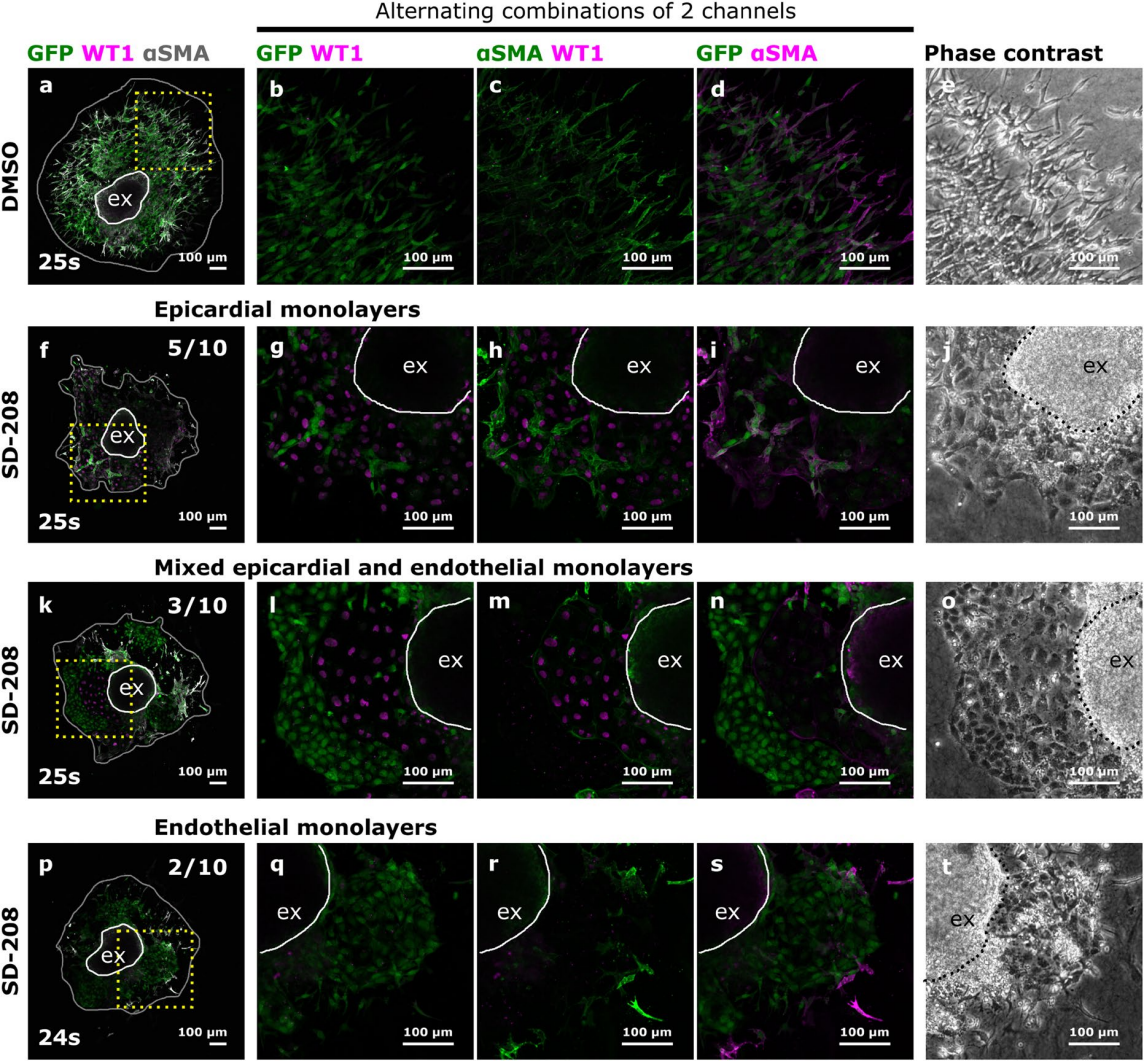
.

In addition, this Article contains an error in the Methods section under subheading ‘Collagen gel preparation’ where

“Rat tail type 1 collagen gels (Merck 08–115) were prepared at 1 mg/ml in 10 × M199 (Life Technologies 11825–015) and the pH was adjusted to 7.5 with 2.2% (w/v) NaHCO3. 10 × M199 and NaHCO3 were both measured at 1/10 of the final volume.”

should read:

“Rat tail type 1 collagen gels (Merck 08–115) were prepared at 1 mg/ml in 10 × M199 (Life Technologies 11825–015) and the pH was adjusted with 2.2% (w/v) NaHCO3. 10 × M199 and NaHCO3 were both measured at 1/10 of the final volume.”

