# Supplementary material for: The epicardium obscures interpretations on endothelial-to-mesenchymal transition in the mouse atrioventricular canal explant assay
Source: Sci Rep. 2018 Mar 16;8:4722. doi: 10.1038/s41598-018-22971-w (PMC5856756; doi:10.1038/s41598-018-22971-w)
Supplement: Supplementary file 1 — Supplementary Figures [file 41598_2018_22971_MOESM1_ESM.pdf]

Supplementary Information

The epicardium obscures interpretations on endothelial-to-mesenchymal transition in the mouse atrioventricular canal explant assay

Nathan Criem[1,2,3] and An Zwijsen[1,2,3]

Figure S1: Somite stage dependent cellular outgrowth in the AVC explant assay

Figure S2: Endothelium-derived mesenchymal cell invasion is maximal in AVC explants of embryos of 20s-23s

Figure S3: SD-208 treatment efficiently inhibits ALK5 activation

Figure S1

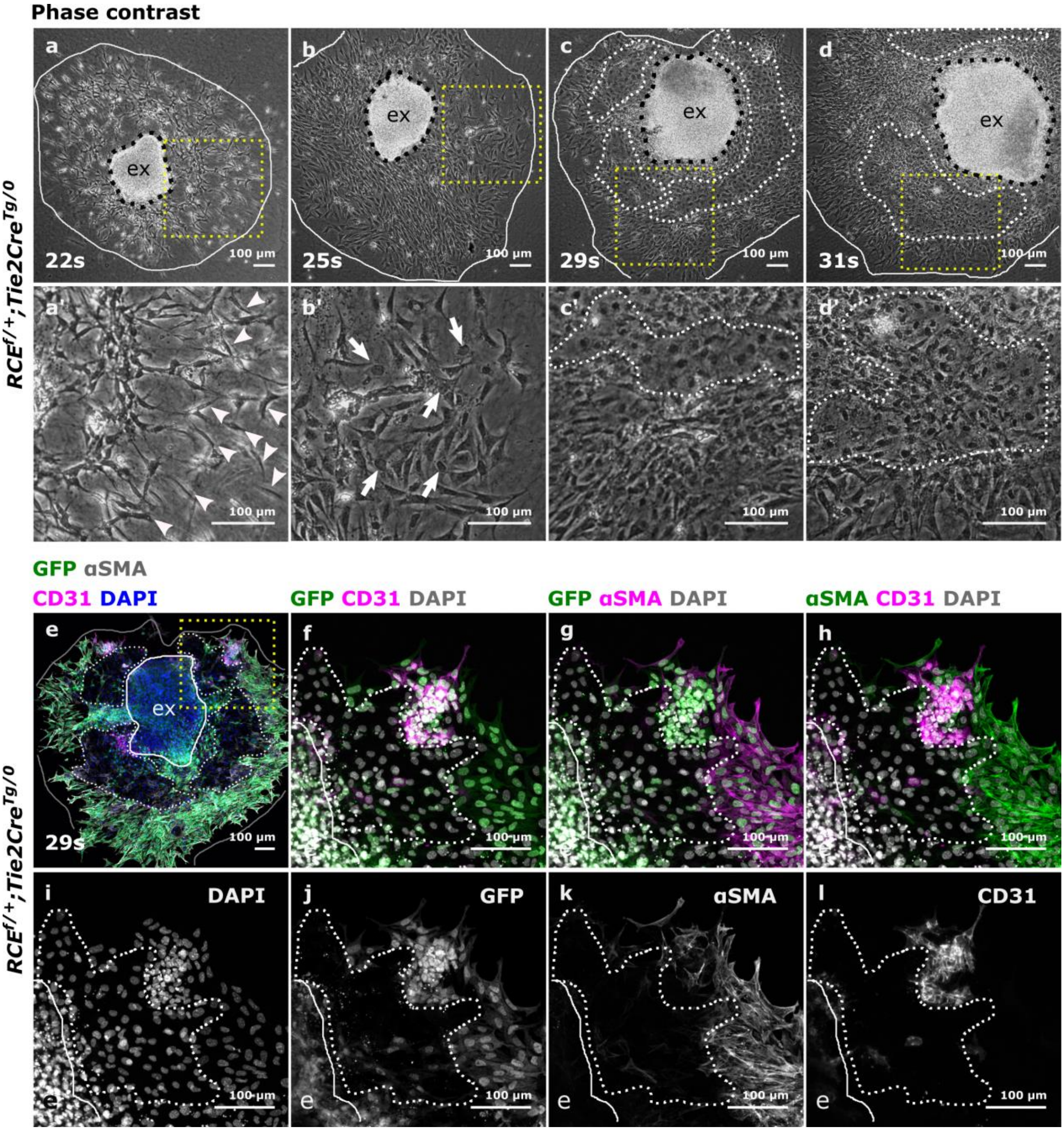

Figure S1: Somite stage dependent cellular outgrowth in the AVC explant assay

(a-d) AVCs from *RCE*$^{f/0}$;*Tie2Cre*$^{Tg/0}$ embryos staged between 20s-36s (N=72 explants) were cultured for 48 and presentative phase contrast images are shown. Boxed areas are magnified in panels (a'-d'). The explant (ex) is delineated by a black dashed loop. Area of cellular outgrowth is delineated by a white loop. The arrowheads in (a') and arrows in (b') represent spindle-like shaped mesenchymal cells and cobblestone-shaped cells respectively.

(e) Overview of an explant stained for GFP, αSMA and CD31. The boxed area in e is magnified in (f-h) and presented as alternating combinations of two channels in magenta and green, overlaid with DAPI in gray. The explant (ex) is delineated by a white loop. The area of cellular outgrowth is delineated by a gray loop.

(i-l) The boxed area in e is separated in the individual channels and represented in gray values. White dashes indicate GFP-negative monolayers.

Figure S2

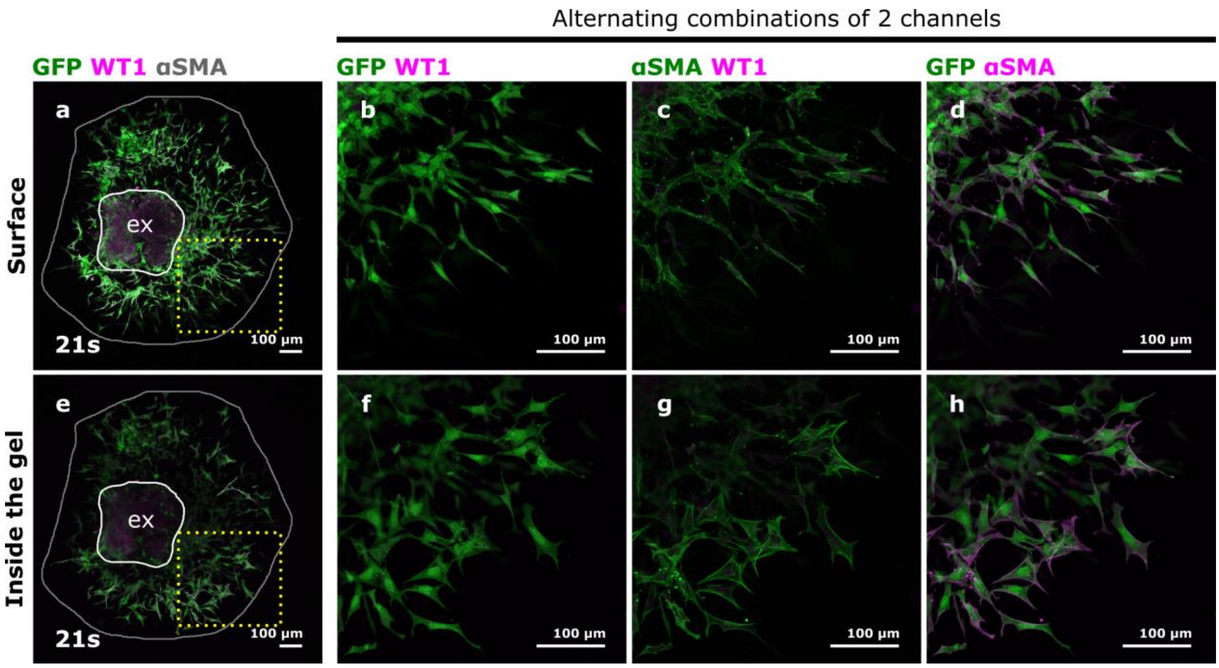

Figure S2: Endothelium-derived mesenchymal cell invasion is maximal in AVC explants of embryos of 20s-23s

Confocal optical sections at the surface of the collagen gel (a) and at approximately 15 μm inside the gel (e) at the same X-Y coordinates of an explant stained for GFP, αSMA and WT1. Boxed areas in panels (a) and (e) are magnified in respectively (b-d) and (f-h) and represented as alternating combination of 2 channels in magenta and green. The explant (ex) is delineated by a white loop. The area of cellular outgrowth in panels (a) and (e) is indicated with a gray loop.

Figure S3

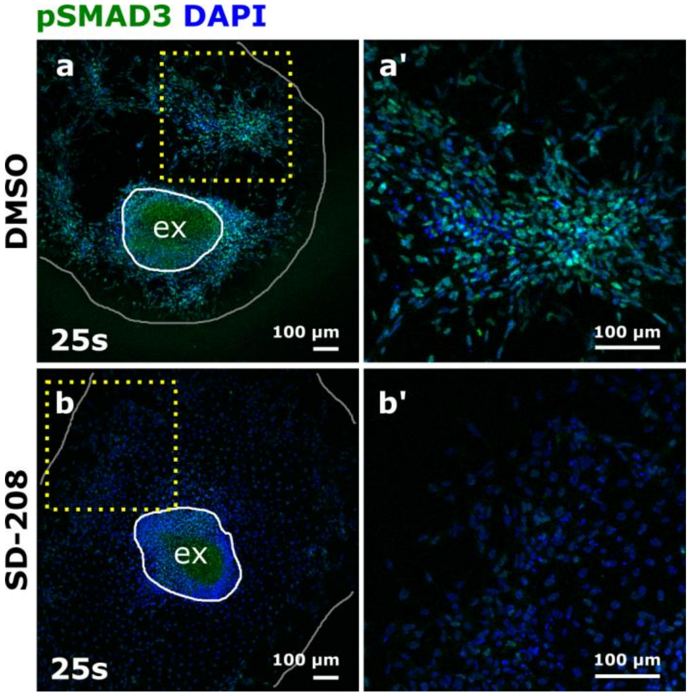

Figure S3: SD-208 treatment efficiently inhibits ALK5 activation

(a-b) AVC explants treated with either DMSO or SD-208 in DMSO were stained for phosphorylated SMAD3 (N=5). Boxed areas are magnified in a'-b'. The explant (ex) is indicated with a white loop. The area of cellular outgrowth is indicated with a gray loop.
